# Battery Electrode Design Process Using Deep Neural Network

**DOI:** 10.1021/acsomega.6c05440

**Published:** 2026-09-28

**Authors:** Hyeonghun Park, Joosoon Lee, Hyungi Song, Kyoobin Lee, Hyeong-Jin Kim

**Affiliations:** † Graduate School of Energy Convergence, 505934 Institute of Integrated Technology, Gwangju Institute of Science and Technology, Gwangju 61005, South Korea; ‡ Department of AI, 65419 Gwangju Institute of Science and Technology, Gwangju 61005, South Korea; § Department of Chemistry, Gwangju Institute of Science and Technology, Gwangju 61005, South Korea

## Abstract

The transition to electric vehicles (EVs) demands lithium-ion batteries (LIBs) customized for diverse energy and power density requirements, yet the intricate trade-offs among electrode design parameters make conventional trial-and-error optimization costly and time-consuming. This study presents the battery electrode exploration network (BattleNet), a deep learning model that predicts the specific capacity across multiple C-rates, collectively representing the rate capability, from electrode design parameters, including loading level, thickness, and porosity. Trained entirely on real-world experimental data, BattleNet achieved an *R* ^2^ of 0.889, outperforming conventional machine learning baselines. Based on these predictions, an inverse design framework was proposed to explore design parameters satisfying a target rate capability. The explored parameters matched the target with an average error of 7.9%, and cells fabricated within the explored region satisfied the target performance with an average error of merely 4.5%. These results demonstrate the practical feasibility of deep learning as a tool to accelerate LIB design and development for the EV industry.

## Graphical abstract

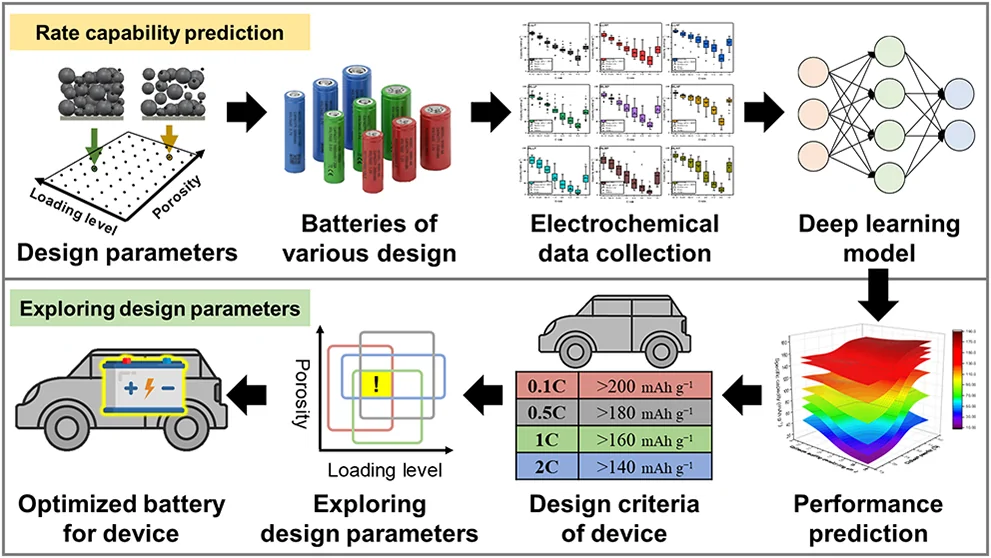

## Introduction

To achieve carbon neutrality by 2050 globally, the transition from internal combustion engines (ICEs) to zero-emission electric vehicles (EVs) is accelerating.^1^ While the initial EV market operated with a limited range of models due to short driving ranges and high prices, the mass production and technological advancement of lithium-ion batteries (LIBs) have recently led to an explosive diversification of EV types, ranging from micro urban vehicles to large commercial trucks.^2^ Consequently, the core battery performances demanded by the market have also been specified and advanced into ‘extended driving range (high energy density)’ and ‘extreme fast charging (high power density)’. However, these two performance metrics have a fundamental trade-off relationship, making it technologically highly challenging to maximize both within a single cell.^3^ As a result, because the weight of required energy and power varies depending on the purpose and size of the vehicle (e.g., short-distance urban driving vs. long-distance freight), it has become more crucial than ever to develop technologies that do not merely aim for a single top-performance battery, but custom-optimize LIB design to meet the diverse specifications and performance requirements demanded by numerous types of EVs.

To realize such demanding customized performance and overcome existing limitations, advanced manufacturing technologies that minimize the tortuosity of lithium ions within the electrode, such as magnetic alignment and 3D electrode structuring, have been actively investigated,^4,5^ alongside efforts to resolve interfacial constraints in solid-state systems.^6,7^ However, due to the strict cost limits and mass-production scalability issues required for EV batteries, the industry still relies heavily on conventional slurry-casting and calendering processes rather than fully adopting these novel technologies immediately. Within this conventional process framework, the most critical challenge for achieving target performance is controlling the theoretical trade-off between ionic and electronic conductivity inside the electrode.^8^ As illustrated in Figure 1a, applying a severe calendering process for fast electronic conduction decreases the porosity of the electrode, thereby significantly sacrificing lithium-ion mobility.^9^

**Figure 1:**
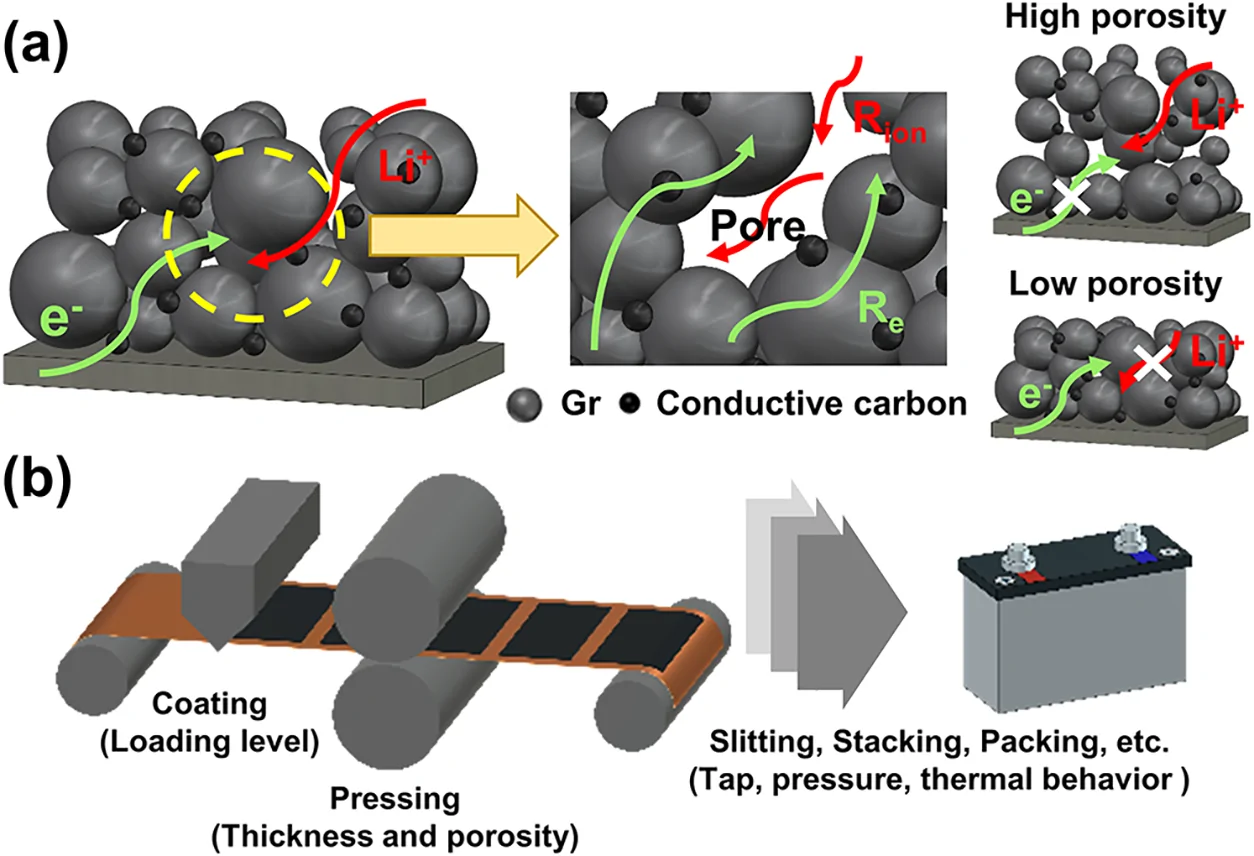
Schematic illustration of (a) the trade–off between electrical and ionic conductivity in the electrode in terms of porosity; (b) manufacturing process of LIB.

Therefore, successful customized electrode design must focus on meticulously tuning various design parameters of the existing process to find the optimal balance of ionic and electronic conductivity required by each EV. As depicted in Figure 1b, these critical parameters extend beyond porosity to include electrode loading level, thickness, tortuosity, composition, and negative-to-positive (N/P) ratio, as well as the positioning and quantity of tabs.^10–12^ Nonetheless, the intricate nonlinear relationships underlying the trade-offs among these individual parameters severely constrain the range of experimental exploration for achieving a balanced optimization between energy and power densities, as necessitated by manufacturing specifications.^13^

More specifically, the impact of each design parameter on the electrochemical performance of LIBs varies nonlinearly depending on operating environments outside the design variables, such as current density and temperature, and abnormal charging conditions.^14^ This makes designing LIBs optimized for predetermined performance criteria even more complex. Despite extensive research spanning decades since commercialization, LIB manufacturers still primarily rely on trial-and-error approaches based on engineering expertise and existing data when developing new batteries. Such traditional design methods require massive human resources and testing time, thereby delaying LIB development and exponentially increasing costs. Thus, the development of data-driven solutions capable of accurately predicting the effect of numerous design parameters on electrochemical performance and actively supporting high-efficiency LIB design is urgently needed.

Recently, artificial intelligence (AI) and machine learning (ML) have been widely adopted to accelerate the development and optimization of LIBs.^15^ Extensive research has focused on predicting performance or cycle life under fixed cell designs. Regularized linear models and Gaussian process regression have been applied to early-cycle voltage features and impedance spectra for cycle life and remaining useful life prediction,^16–18^ and Bayesian optimization has been combined with early prediction to identify optimal fast-charging protocols.^17^ Gradient boosting regression has predicted cathode discharge capacity from compositional descriptors.^19^ More recently, deep learning frameworks have generalized lifetime prediction across diverse aging conditions,^20,21^ GPT-based models have predicted full degradation trajectories from early data,^22^ and physics-informed neural networks have improved degradation prognosis.^23,24^ Formation data have also been leveraged for pre-deployment quality classification.^25^ However, these studies address active material properties^19^ or cycle life of cells with predetermined designs,^16–18,20–26^ without considering electrode manufacturing parameters that govern cell performance.

Studies optimizing electrode design parameters using AI have also emerged. Surrogate models trained on synthetic data from physics-based simulations coupled with electrochemical models have been used for bi-objective optimization of energy and power density,^27–29^ and simulation-coupled machine learning approaches have optimized gradient porosity and thick electrode rate capability.^30–32^ Experimentally, ML has been applied to coating process data^33^ and electrode cross-section images^34^ to model process–performance relationships. However, most works rely on virtual data from physics-based simulations, and the few experimental studies either address only individual manufacturing steps such as coating^33^ or use indirect inputs such as cross-section images,^34^ rather than directly mapping electrode design parameters to electrochemical performance across a wide range of C-rates, which fundamentally governs the energy–power density trade-off.

To overcome these limitations, this study focuses on the optimization of actual manufacturing parameters based entirely on real-world experimental data rather than virtual simulations. We developed the battery electrode exploration network (BattleNet), an MLP-based deep learning model that extracts and fuses features from the electrode design parameters (loading level, thickness, and porosity) of both electrodes to predict the specific capacity across multiple C-rates, collectively representing the rate capability. BattleNet achieved an *R* ^2^ of 0.889, outperforming conventional machine learning baselines, and an inverse design framework built on these predictions explored design parameters tailored to a target capacity, which was experimentally validated with an average error of merely 4.5%. This study demonstrates the potential of deep learning as a practical tool to advance the LIB industry by significantly reducing the time and effort required for development.

## Experimental Details

### Preparation of Electrode and Cell Assembly

The cathode slurry, consisting of 93 wt % NMC811 (L&F), 4 wt % Super-C (MTI Co.), and 3 wt % poly­(vinylidene fluoride) (Solef 5130, Solvay) in *N*-methyl pyrrolidone, was coated on aluminum foil (30 μm, 8.15 mg cm^–2^), and the anode slurry, consisting of 96 wt % graphite (Hitachi), 1 wt % Super-P, 1.5 wt % carboxymethyl cellulose (Sigma-Aldrich), and 1.5 wt % styrene–butadiene rubber (MTI Co.), was coated on copper foil (30 μm, 22.38 mg cm^–2^), each at various areal loading levels. Electrodes with varying loading densities were calendered to distinct thicknesses to yield a range of porosities. The mass and dimensions of these electrodes were quantified using XSR204 V (Mettler Toledo) for weight and VL-50 (Mettler Toledo) for thickness. Field emission scanning electron microscopy (FE-SEM, JSM-7500F, JEOL) was employed to examine the cross-sectional morphology of the electrodes. For each paired condition, the electrode discs were punched from a single coated and calendered sheet, so that replicate cells shared the same fabrication condition, and the cathode and anode were paired by matching their loading levels to target a designed N/P ratio of ∼1.1. A reference electrolyte was formulated using ethylene carbonate and diethyl carbonate in a 1:1 volume ratio, combined with 1 M LiPF_6_ (SoulBrain Co.) serving as the lithium salt. NMC811 cathodes were fabricated into discs with a 10 mm diameter, while graphite anodes were shaped to a 14 mm diameter to mitigate the formation of lithium dendrites on the copper foil. Following punching, all electrodes were dried at 60 °C for 24 h in a vacuum oven before assembly.

All 2032 coin cells (Wellcos Co.) were assembled in an argon–filled glove box (Mbodytech, H_2_O < 1 ppm) with 50 μL electrolyte. A polypropylene Celgard 2400 separator and stainless steel spacer with a thickness of 1.3 mm were used. A total of 247 cells were assembled by manufacturing 27–33 cells for each pair. Following assembly, all the cells were maintained at 25 °C for 12 h to facilitate electrolyte penetration.

### Electrochemical Data Collection and Pre-Processing

The cells were charged and discharged at 2.8–4.3 V to measure the rate capability. The current density was selected from the range from 0.1 to 5C in terms of commercial interest^35^ where 1C is the current that charges a battery in 1 h. The charging process consisted of a constant–current (CC) phase followed by a constant–voltage (CV) phase at 4.3 V, which was maintained until the current decreased to one-tenth of the applied charging C–rate. After a 10–minute rest period, the cells were discharged under a CC protocol to 2.8 V at the corresponding C–rate. Electrochemical assessments were carried out within a thermostatic chamber set at 25 °C to regulate the cell temperature, irrespective of the heat produced during functioning. A galvanostatic battery cycler (WBCS 3000, WonATech) served to evaluate the electrochemical performance of the NMC811/graphite full cell. To ensure data reliability, this charge–discharge cycle was repeated five times for each of the seven different current densities, and the specific capacity was determined by averaging these five measurements, generating a total of 1729 data points. To our knowledge, this is the first publicly available dataset for LIBs with varying designs of electrodes and current density.

### Rate Capability Prediction Model

BattleNet predicts the specific capacities across seven distinct C–rates from the design parameters of LIBs, as illustrated in Figure 2. The model extracts features from two complementary views in parallel. In the electrode-wise branch, the design parameters of the cathode and those of the anode (each in *R* ^3^) are processed by dedicated feature extractors and fused into an interelectrode feature (*R* ^1024^). In the parameter-wise branch, the paired loading levels, porosities, and thicknesses of the two electrodes (each in *R* ^2^) are processed in the same manner and fused into an interparameter feature (R^1024^). Each feature extractor is a three–layer multilayer perceptron (MLP), and each fusion module concatenates its inputs and processes them through another three–layer MLP. The interelectrode and interparameter features are then combined into a final fusion feature (*R* ^1024^), from which dual prediction heads output the specific capacities at low C–rates (0.1, 0.2, 0.5, and 1C) and high C–rates (2, 3, and 5C), respectively.

**Figure 2:**
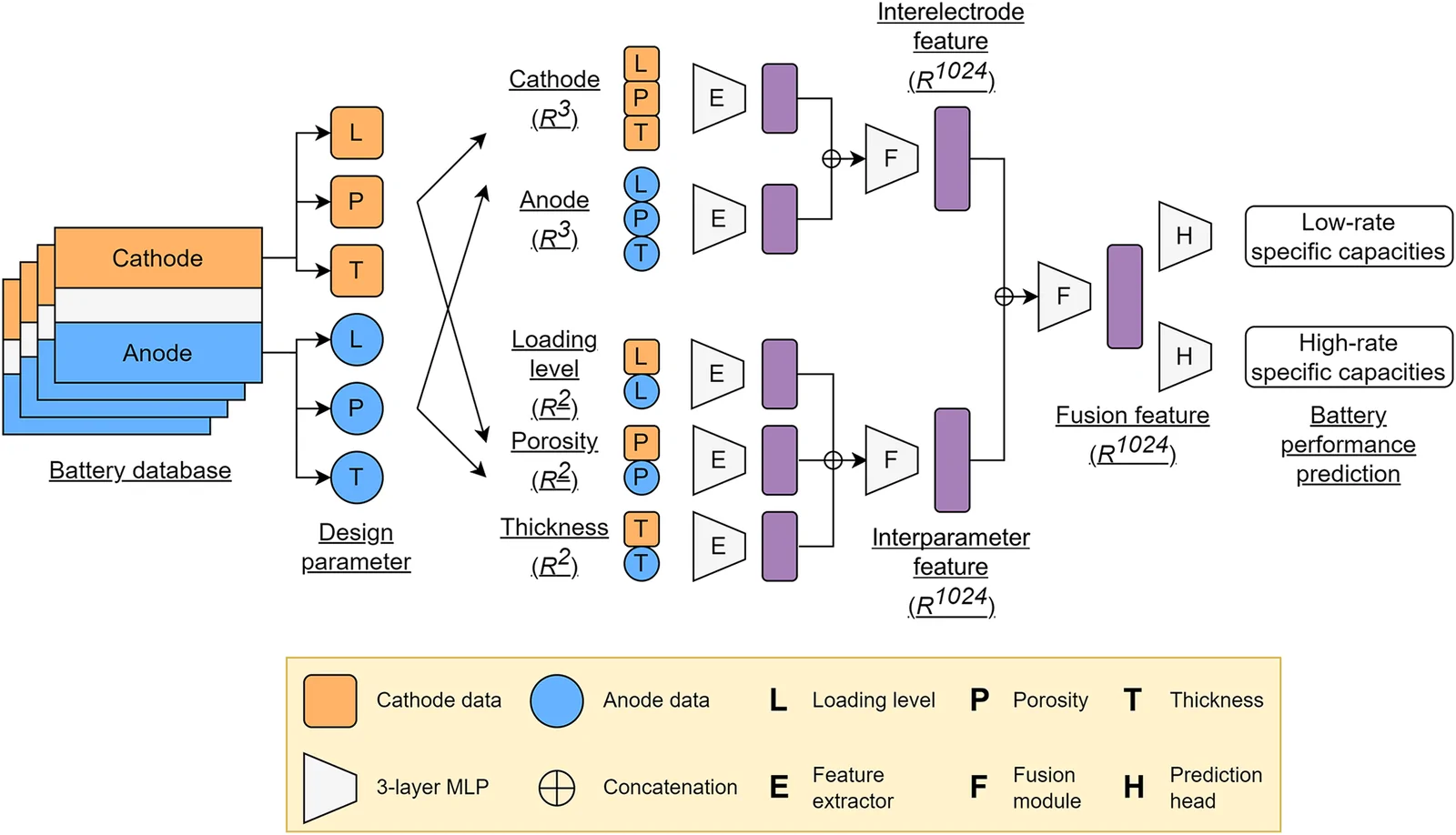
Architecture of the proposed rate capability prediction model.

BattleNet was implemented in the PyTorch framework and trained using the L2 loss and Adam optimizer, along with a learning rate of 0.001. The training required approximately 20 min during 500 epochs on an RTX 3090 GPU.

### Inverse Design Framework for Exploring LIB Design Parameters

The inverse design framework for exploring LIB design parameters that satisfy target performance is illustrated in Figure 3. First, synthetic battery candidates were generated by sampling design parameters within the range of the dataset constructed in this work. The loading level and porosity of the cathode were sampled in increments of 0.3 mg cm^–2^ and 0.5%, respectively. The porosity of the anode was sampled in increments of 0.5%, and its loading level was sampled at four N/P ratios (1.0, 1.1, 1.2, and 1.3) relative to the cathode loading level. The trained BattleNet then predicted the rate capability of each synthetic candidate, and only those whose predicted specific capacity exceeded the target at all C-rates were selected.

**Figure 3:**
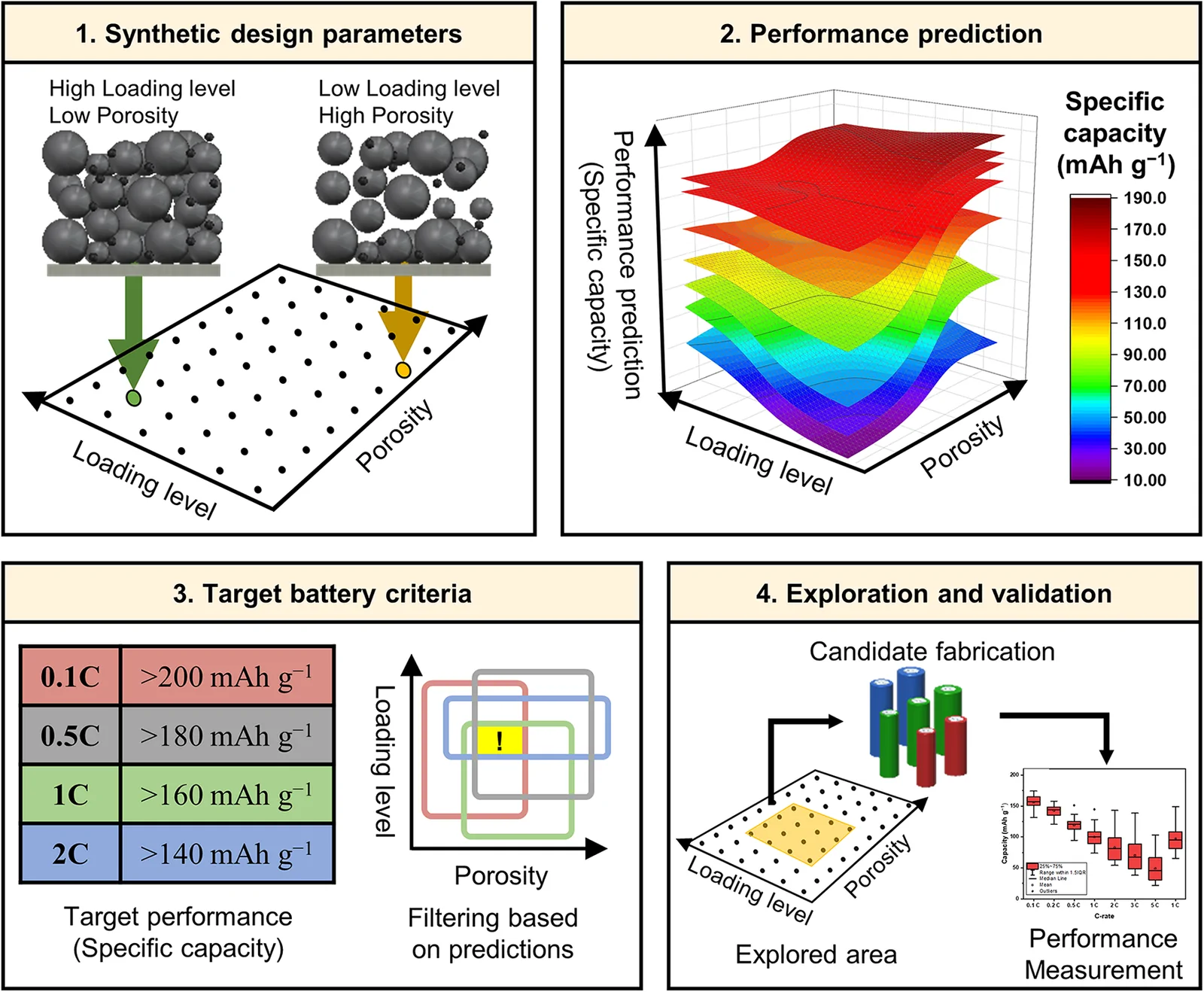
Schematic illustration of the design parameter exploring framework using the battery performance prediction model.

To evaluate the exploration accuracy, the measured performance of each cell in the 6-fold cross-validation test set was used as the target, and the framework explored design parameters accordingly. Since multiple parameter combinations can satisfy a given target, the framework returns an explored region rather than a single candidate. Therefore, the closest point in the explored region to the measured value was identified using Euclidean distance (eq 1), and the percentage error between them was calculated as the search error (Figure S1). In addition, cells were fabricated using the explored design parameters, and their measured capacities were compared against the predicted values to validate the practical feasibility of the framework.

1 $$\mathrm{Euclidean}\;\mathrm{distance}=\sqrt{\sum_{i=1}^{N}{\left({p\mathrm{arameter}}_{m\mathrm{easured},i}-{p\mathrm{arameter}}_{p\mathrm{rediction},i}\right)}^{2}}$$

## Results and Discussion

### LIBs Data Analysis

The study included the construction of cells using different electrode design parameters to measure the electrochemical performance and produce a dataset. We fabricated 247 cells by varying the major design parameters of electrode loading level, thickness, and porosity (Figure 4a,b).^36–39^ Cross–sectional FE–SEM imagery of both the cathode and anode (Figures S2 and S3) confirmed the successful fabrication of electrodes with varied design configurations. Specifically, the microscopic pore structure observations reveal that heavily calendered electrodes with low porosity exhibit densely packed active materials. Such constrained interstitial spaces severely restrict the mobility of lithium ions within the electrolyte during fast charge–discharge (high C-rate) conditions, which can lead to a rapid increase in concentration polarization. Subsequently, the specific capacities of cells fabricated with these diverse electrodes were quantified across seven distinct current densities, thereby generating a comprehensive dataset. This dataset encompasses rate capability, which serves as a foundational elements for computing energy- and power- densities. The dataset underwent rigorous analysis to validate the presence of a highly nonlinear region that parameterizes the capacity of the LIBs. Initially, the influence of individual parameters on the (dis)­charge voltage of the cell was ascertained, as shown in Figure 4d–f. Relative to alterations in discharge voltage, fluctuations in charge voltage exhibit a more pronounced impact, attributable to the higher resistance encountered during the insertion of lithium ions into the anode compared to the cathode.^40^ As the loading level and thickness increased and the porosity decreased, the resistance to the movement of lithium ions through the pores increased,^41,42^ enlarging the difference between the (dis)­charge voltages.

**Figure 4:**
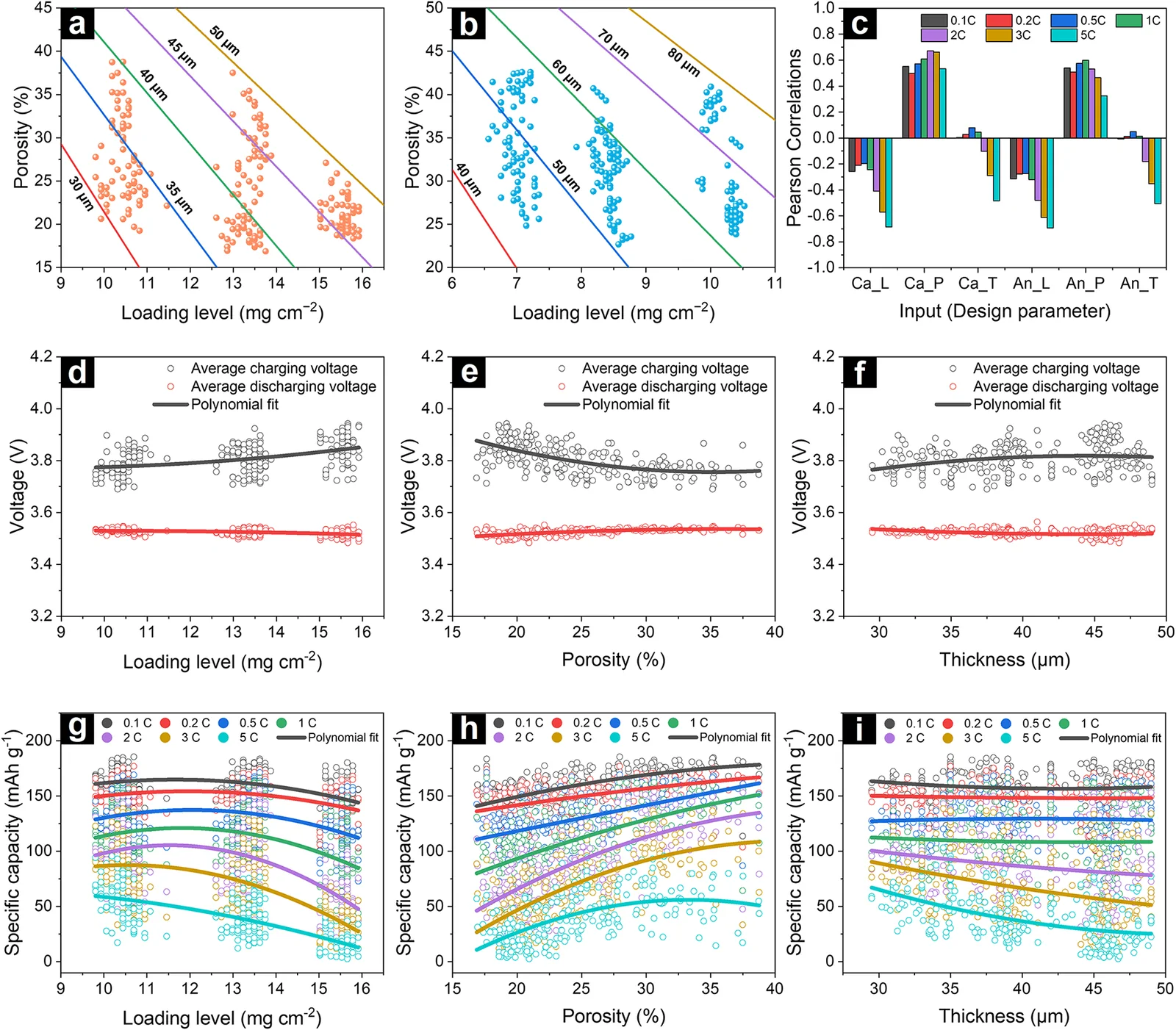
Electrode designs of the training and test sets of the (a) NMC811 cathode and (b) Gr anode. (c) Pearson correlation coefficient for each parameter according to the rate capability. Correlation between the electrochemical performance and (d, g) loading level, (e, h) porosity, and (f, i) thickness with the (d–f) average voltage and (g–i) specific capacity.

A trend of diminishing specific capacity is observed in correlation with rising resistance, as evidenced in Figures 4g–i. Concurrently, variations in specific capacity attributable to design parameters manifest differentially depending on the current density involved. Such variations are ascribed to the influence of electrochemical kinetics at lower current densities, particularly on the charge–transfer reaction. In contrast, the diffusion of lithium ions in the electrolyte dominates the electrochemical kinetics as the current density increases.^43^

For a more nuanced analysis, the Pearson correlation coefficient was computed for each design parameter in relation to their rate capability, as shown in Figure 4c. Ca, An, L, P, and T mean cathode, anode, loading level, porosity, and thickness, respectively. All the correlation coefficients between the design parameters and the specific capacity of the electrode varied depending on the current density. The relatively dense distribution for porosity is shown since the two effects cancel each other out because increasing thickness concerning the same loading level increases porosity, and increasing loading level with respect to the same thickness reduces porosity. Nevertheless, the Pearson correlations of Ca_L, Ca_T, An_L, and An_T significantly vary at 2C. This phenomenon can be physically explained by the transition in the rate-determining step of the cell. At low C-rates, the cell performance is primarily governed by charge transfer and electronic conductivity. However, at higher C-rates (≥2C), the system approaches the diffusion-limited C-rate (DLC),^44^ where the mass transport of lithium ions within the solid and liquid phases becomes the dominant limiting factor. Beyond this threshold, the restricted lithium diffusion within the narrowed pore network leads to a nonlinear dependence on structural parameters like porosity and thickness, causing the observed abrupt shift in the correlation. Inspired by this correlation, high (2, 3, and 5C) and low (0.1, 0.2, 0.5, and 1C) rate capabilities are separately predicted.

The findings underscore the imperative for systems endowed with robust computational capabilities to navigate the intricate landscape of design parameters. Specifically, Figures S4–S7, illustrating diverse electrochemical performances as influenced by both loading level and porosity, highlight the escalating complexity in discerning underlying trends with the addition of more parameters. Furthermore, visual representation of these trends becomes unfeasible when the count of parameters exceeds four, as indicated in Figure S8. Given the constrained predictability inherent in complex systems—amplified by fluctuating correlations emerging as response kinetics vary with design parameters—the task of feature extraction for subsequent incorporation into machine learning models poses considerable challenges. Therefore, deep learning techniques based on the datasets were introduced to enhance the predictive capability significantly.

### Rate Capability Prediction

The rate capability prediction performance of the proposed model, BattleNet, was evaluated on the collected LIB electrochemical dataset and compared with machine learning regression baselines (ridge, lasso, elastic net, decision tree, and random forest). All models were evaluated via 6-fold cross-validation repeated five times with different random seeds, and the MAE, RMSE, and *R* ^2^ (eqs 2–4; lower MAE and RMSE and higher *R* ^2^ indicate better performance) averaged over all runs are summarized in Table 1.

2 $$\mathrm{RMSE}=\sqrt{\frac{1}{n}\sum_{i=1}^{n}{\left({ŷ}_{i}-y_{i}\right)}^{2}}$$

3 $$\mathrm{MAE}=\frac{1}{n}\sum_{i=1}^{n}\left|{ŷ}_{i}-y_{i}\right|$$

4 $$R^{2}=1-\frac{\sum_{i=1}^{n}{\left(y_{i}-{ŷ}_{i}\right)}^{2}}{\sum_{i=1}^{n}{\left(y_{i}-\mathrm{y̅}\right)}^{2}}$$

where *n* is the total number of data points, *y*_*i*_ is the ground truth of the measured value, *ŷ*_*i*_ is the predicted value from the model, and *y̅* is the mean value of all the *y*_*i*_.

**Table 1:** Specific Capacity Prediction Results^a^

model	MAE (mAh g^–1^)	RMSE (mAh g^–1^)	*R* ^2^
Ridge	13.7	17.9	0.859
Lasso	14.0	18.2	0.855
Elastic net	14.2	18.5	0.850
Decision tree	15.6	20.8	0.810
Random forest	13.2	17.6	0.865
**BattleNet (ours)**	**12.1**	**15.9**	**0.889**

a The proposed method, BattleNet, is compared with machine learning regression baselines.

BattleNet exhibited remarkable accuracy in predicting the measured rate capability as well as the loci of inflection points based on design parameters, as showcased in Figure S9. For a rigorous quantitative assessment, BattleNet’s performance was compared with other machine learning algorithms. The scatterplots in Figures 5a–d and S10a,b show the correlation between the predicted and empirically measured specific capacities for each modeling approach. In these plots, the black linear trajectory signifies the measured specific capacities, while the individual scatter points denote predicted values. A red linear trendline serves as a linear fit function for these scattering data points. In comparison to traditional machine learning models, both the scatterplot and the fit function for BattleNet closely align with the equation *y* = *x*, as highlighted in the inset histogram. This suggests that BattleNet surpasses benchmark models in prediction precision. Additionally, BattleNet maintains a low error across an expansive range of loading levels and porosities, as evidenced in Figures 5e–h and S10c,d. In these contour plots, the maximum and minimum error values are explicitly annotated for intuitive comparison. Notably, BattleNet exhibits the lowest error standard deviation (13.31 mAh g^–1^) compared to the baselines (15.55–17.28 mAh g^–1^), indicating that its errors are more densely concentrated around zero. Collectively, these findings confirm the superior precision and fidelity of the deep learning model in deciphering the complex nonlinear dynamics inherent in LIB systems.

**Figure 5:**
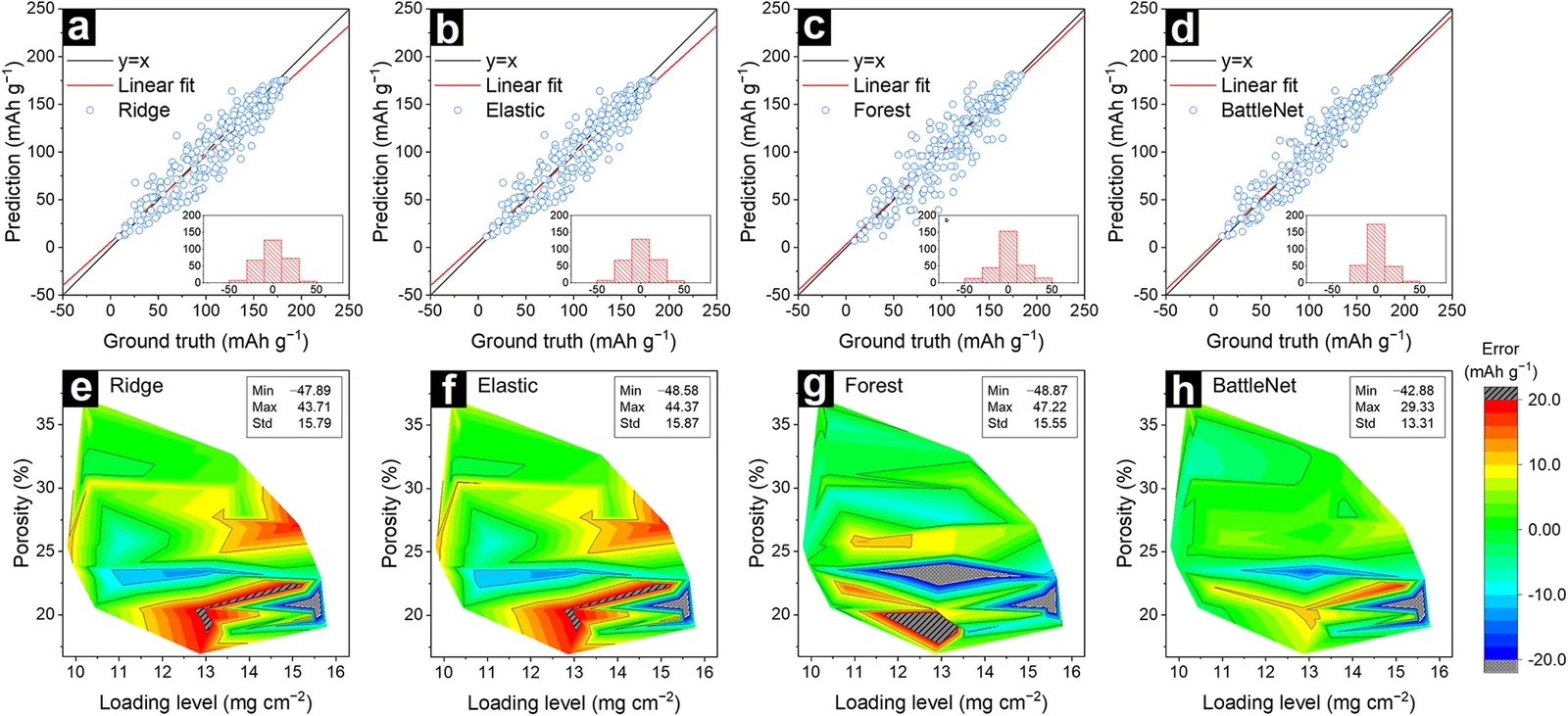
(a–d) Measured vs. predicted capacities for test sets and (e–h) errors based on the loading level and porosity obtained using (a, e) ridge regression, (b, f) elastic net regression, (c, g) random forest regression, and (d, h) BattleNet.

To validate the contribution of each input parameter, an ablation study was conducted by excluding one of the three design parameters—loading level, thickness, or porosity—and retraining BattleNet under the same 6-fold cross-validation protocol. As shown in Table 2, the full three-parameter model outperformed all ablated configurations. Removing porosity led to the largest performance drop, which aligns with its known role in the ionic and electronic transport balance within the electrode. Although these parameters are physically interrelated—porosity can be derived from loading level and thickness—including all three improved prediction accuracy. This indicates that the feature extraction and fusion architecture of BattleNet utilizes the interdependencies among correlated parameters as complementary inputs.

**Table 2:** Ablation Study on Input Parameter Combinations for Specific Capacity Prediction^a^

input data combination					
loading level	thickness	porosity	MAE (mAh g^–1^)	RMSE (mAh g^–1^)	*R* ^2^
√	√		14.6	19.0	0.840
√		√	13.3	17.5	0.867
	√	√	13.5	17.7	0.863
√	√	√	**12.1**	**15.9**	**0.889**

a A checkmark (√) indicates that the corresponding parameter was included as input data.

The architecture of BattleNet was further validated by an ablation study on the configurations of the feature extractor and the capacity prediction head, conducted under the same 6-fold cross-validation protocol (Table 3; the variant architectures are illustrated in Figure S11). For the feature extractor, the proposed configuration, which extracts electrode-wise and parameter-wise features in parallel, was compared with a single extractor processing all input parameters together (Extr-All) and with each decomposition used alone (Extr-Electr and Extr-Param). The proposed extractor outperformed all ablated configurations, and notably, both single-view configurations performed worse than even the unified extractor, indicating that partitioning the inputs from a single perspective discards interactions across the partition, such as that between the cathode loading level and the anode porosity. By combining the two complementary decompositions and recombining them through the fusion modules, the proposed extractor captures the detailed features of each electrode and each parameter while preserving the interactions among all inputs.

**Table 3:** Ablation Study on the Configurations of the Prediction Head and the Feature Extractor for Specific Capacity Prediction^a^

model	feature extractor	prediction head	MAE (mAh g^–1^)	RMSE (mAh g^–1^)	*R* ^2^
**BattleNet (ours)**	Electrode/parameter-wise	Low/high-rate split	**12.1**	**15.9**	**0.889**
Extr-All	Unified	Low/high-rate split	12.6	16.1	0.881
Extr-Electr	Electrode-wise	Low/high-rate split	12.9	16.5	0.875
Extr-Param	Parameter-wise	Low/high-rate split	12.8	16.4	0.877
Head-All	Electrode/parameter-wise	Unified	15.0	18.8	0.831
Head-Indiv	Electrode/parameter-wise	Per-C-rate	12.7	16.3	0.878
Head-Rand	Electrode/parameter-wise	Random split	14.5	18.3	0.840

a All variants were trained and evaluated under the same 6-fold cross-validation protocol.

For the prediction head, the proposed low/high-rate split was compared with a single head predicting the specific capacities at all seven C–rates (Head-All), individual heads each predicting the capacity at a single C–rate (Head-Indiv), and heads covering randomly grouped C–rates (Head-Rand). Replacing the low/high-rate split with a single head led to the largest performance drop among all tested variants. This degradation is attributed to the abrupt change in the parameter–capacity relationship between 1C and 2C identified in the correlation analysis (Figure 4c): as the dominant kinetics shift from the charge–transfer reaction to lithium-ion diffusion in the electrolyte, a single head is forced to fit two conflicting mappings with shared output weights. Head-Rand, which divides the C–rates without regard to this kinetic boundary, recovered only a small portion of the drop, indicating that the improvement originates from aligning the grouping with the kinetic transition rather than from dividing the output itself. Head-Indiv employs the largest number of heads among all configurations, yet performed worse than the proposed two-head split, showing that enlarging the model does not guarantee higher accuracy and that grouping the C–rates dominated by the same rate-limiting process allows each head to learn the characteristics shared across the grouped rates.

### Inverse Exploration of LIB Design Parameters

To visualize the predictive behavior of BattleNet (Figure 6), 500,000 synthetic LIBs were generated with sampled design parameters, and their specific capacities were predicted. For three-dimensional visualization, the cathode design parameters were varied while the anode parameters were fixed at an N/P ratio of 1.24 and a porosity of 32.5%. The predicted specific capacity varies with both current density and design parameters. At 0.1C, cells with high porosity (>40%) and high loading levels yield the highest specific capacity, whereas at higher C-rates, cells with moderate porosity (∼35%) and low loading levels exhibit superior performance, reflecting the nonlinear trade-offs in LIB systems, consistent with experimentally observed cell behavior.

**Figure 6:**
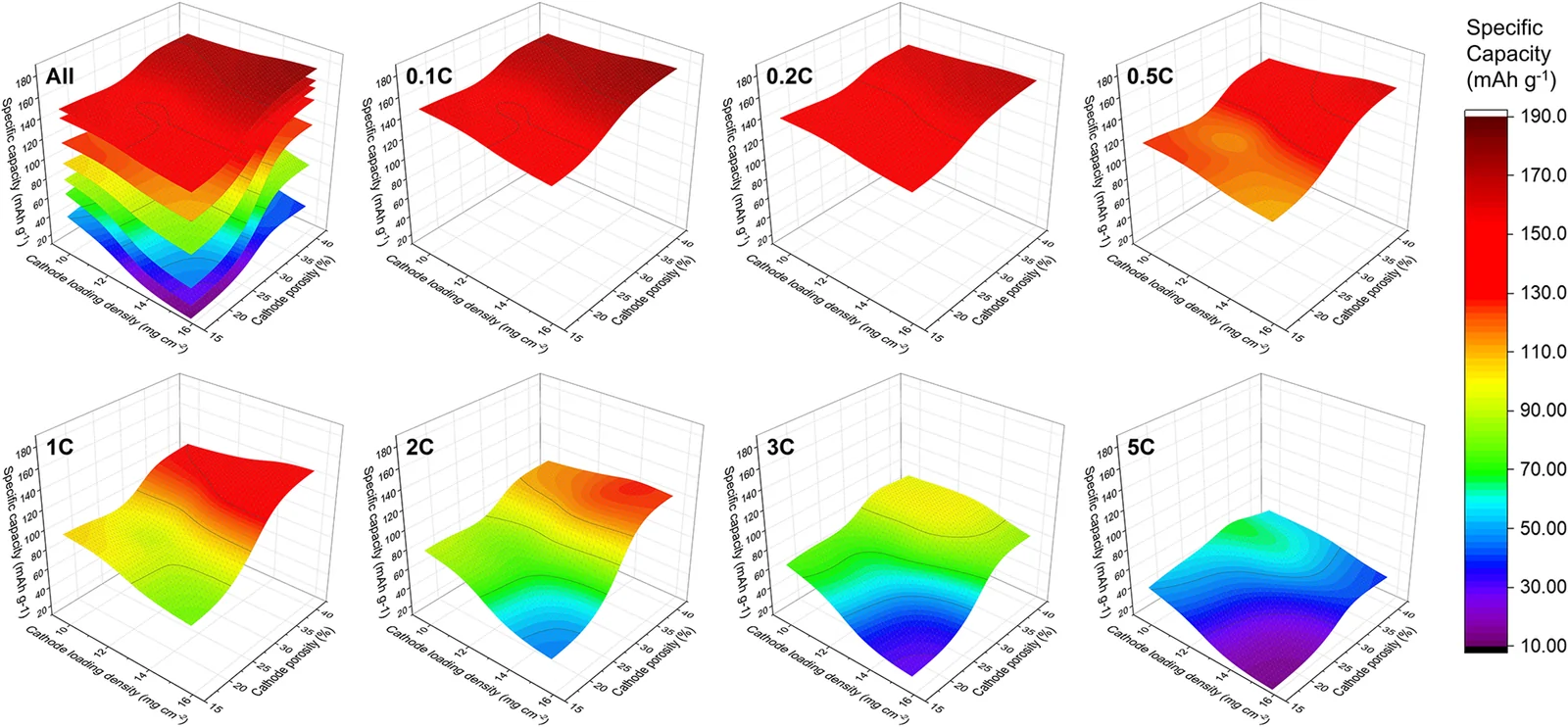
Predicted capacity profiles corresponding to the design parameters through the deep learning model.

The exploration framework was evaluated following the protocol described in the Experimental Details, and the search errors averaged across all folds are presented in Table 4; the search errors ranged from 6.6% to 10.2% depending on the parameter, with an overall average of 7.9%. Figure 7 shows examples of the explored and measured design parameters, with the visualization focused on cathode loading level and porosity. Red circles indicate the measured design parameters from the test set, and the yellow regions represent the explored parameter space predicted to satisfy the target performance. The exploration was completed in approximately 10 min of computation.

**Figure 7:**
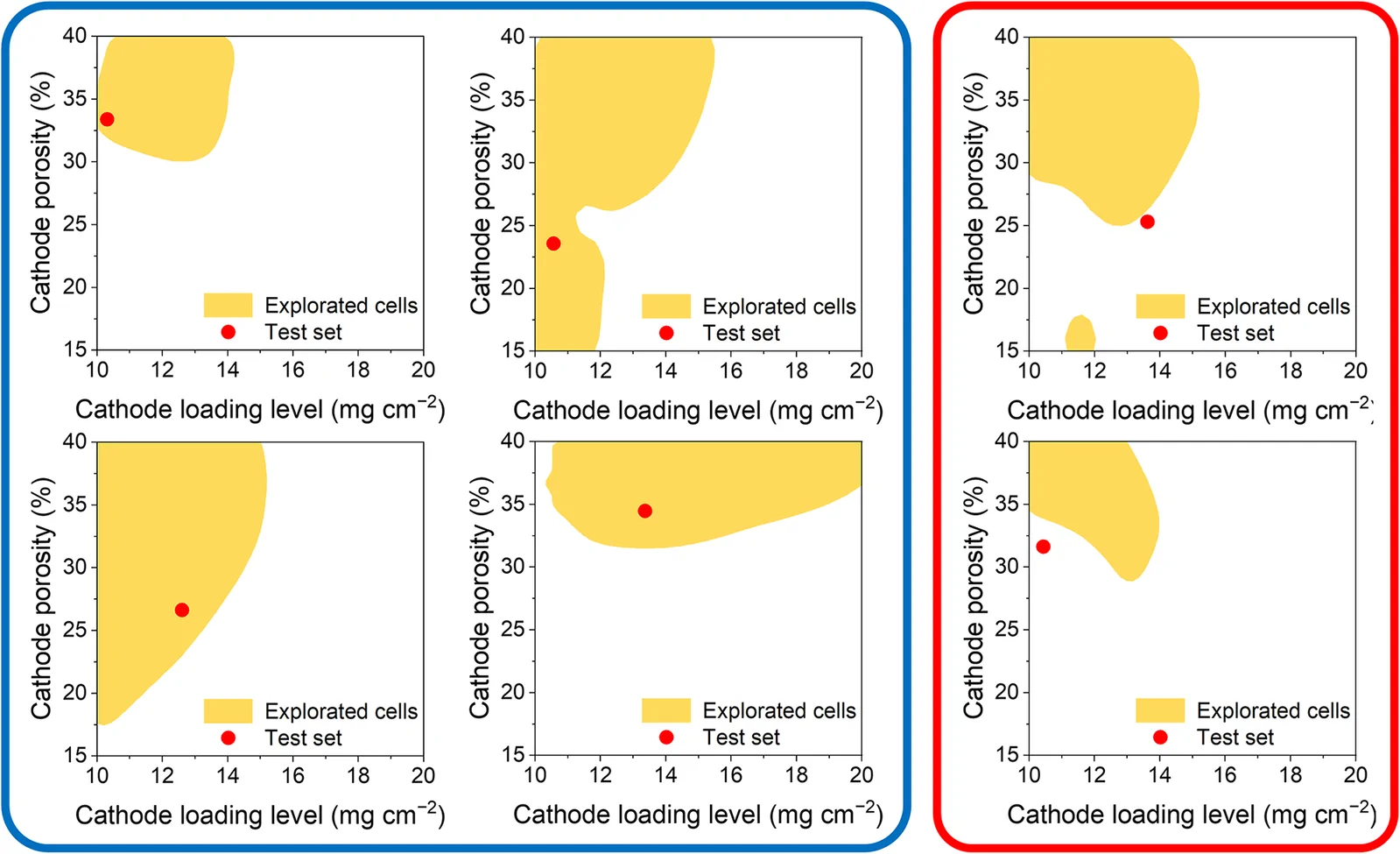
Exploration examples visualized with loading level and porosity of the cathode; blue box: included, red box: not included.

**Table 4:** Search Error of the Proposed Design Parameter Exploration Framework Using BattleNet

	cathode			anode			
design parameter	loading level	porosity	thickness	loading level	porosity	thickness	average
Search error [%]	8.2	7.6	10.8	6.9	6.6	7.5	7.9

To experimentally validate the framework, an arbitrary target rate capability was set and the corresponding design parameter space was explored. It should be noted that the identified designs (Table S1) correspond to previously untested parameter combinations; each design parameter lies within its experimentally sampled range, and the successful fabrication and validation of these cells indicate that the framework can generalize beyond the fabricated design groups rather than merely reproducing sampled configurations. Four cells were randomly selected from the explored region, fabricated (Figure 8a), and electrochemically tested, with their detailed designs listed in Table S1. Figure 8b and Table S2 compare the target and measured rate capabilities of the fabricated cells. Since the framework aims to find designs that meet or exceed the target, only cases where the measured capacity fell below the target were counted as errors (Table S3). The average percentage error across all C-rates was 4.5%, confirming the practical feasibility of the inverse design framework.

**Figure 8:**
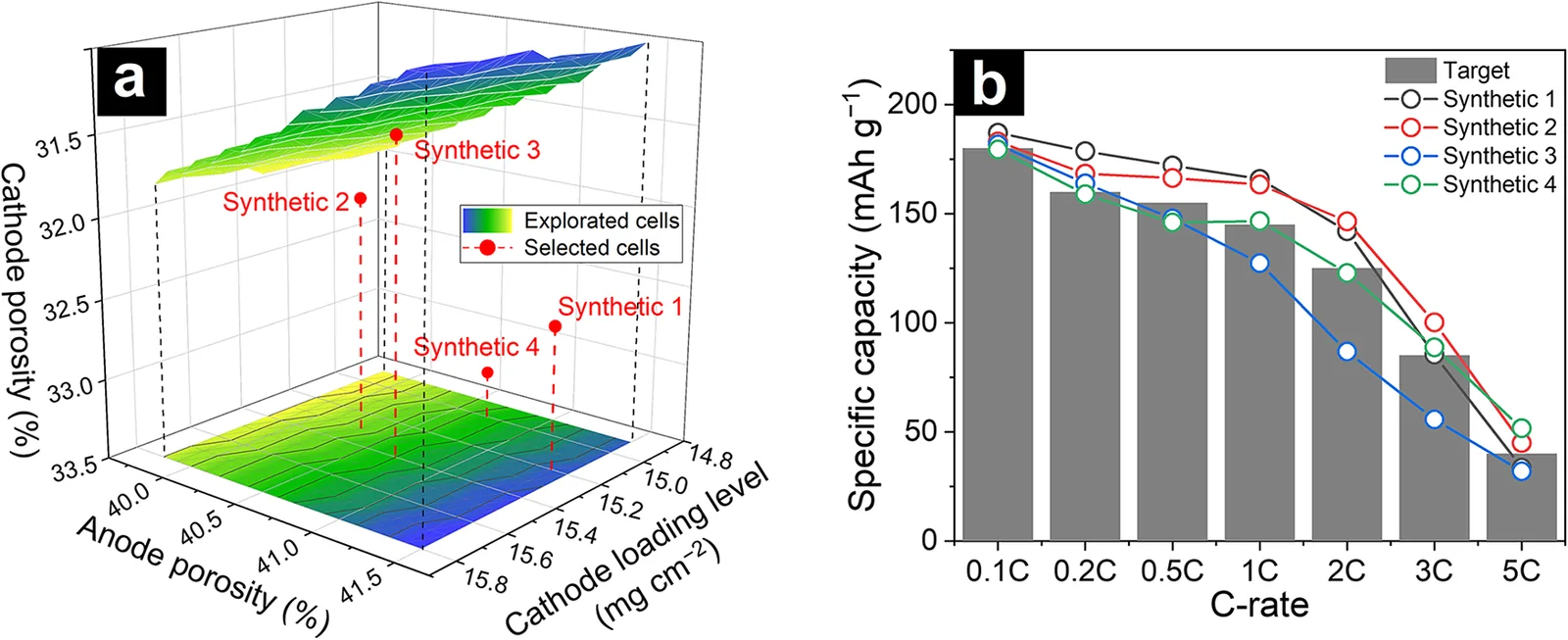
Cell fabrication and electrochemical test based on the reverse design. (a) Explored design parameter space satisfying the target rate capability. (b) Rate performance of the cell randomly selected in the explored space.

### Current Limitations

The present study has several limitations. First, only three design parameters (loading level, porosity, and thickness) were incorporated into the model due to the laboratory-scale scope of this study. However, LIB manufacturing involves numerous additional parameters, including the type of active material, electrode composition, and the electrode-to-electrolyte ratio. Ensuring the model’s robustness when incorporating these additional variables remains a challenge.

Additionally, the dataset was limited to 247 cells fabricated using pilot-scale equipment, which may introduce manufacturing-related deviations compared to industrial-scale production. Expanding the dataset in both size and diversity of design parameters would further improve the model’s predictive accuracy and generalizability.

## Conclusions

In this study, we developed BattleNet, a deep learning model that predicts the rate capability of LIBs from electrode design parameters (loading level, porosity, and thickness). A dataset of 247 cells with varied electrode designs was constructed and used for training. BattleNet predicted the rate capability with an MAE of 12.1 mAh g^–1^, RMSE of 15.9 mAh g^–1^, and *R* ^2^ of 0.889, outperforming conventional machine learning methods.

Based on these predictions, an inverse design framework was proposed to explore electrode design parameters satisfying a target rate capability. The framework achieved an average search error of 7.9%, and cells fabricated within the explored region satisfied the target with an average percentage error of 4.5%. The entire exploration process required approximately 10 min of computation, enabling rapid evaluation of candidate designs without physical cell fabrication.

While this study was limited to three design parameters, incorporating additional manufacturing variables—such as electrolyte ionic conductivity, carbon material conductivity, and active material type and morphology—could further improve prediction accuracy. The influence of cell form factor on electrochemical performance also warrants investigation given the diversifying demands of the EV market. We expect that AI-driven design frameworks such as the one presented here will reduce the time and cost of LIB electrode development.

## Supplementary Material



## Data Availability

The data and code that support the findings of this study are openly available on GitHub at https://github.com/joosoon/battery.

## Notes

Schematic of the search error calculation (Figure S1); cross-sectional FE-SEM images of the cathode and anode (Figures S2 and S3); electrochemical performance as a function of loading level and porosity (Figures S4–S7); visualization of design parameter trends (Figure S8); BattleNet rate capability prediction curves (Figure S9); benchmark comparison plots (Figure S10); detailed designs of the fabricated cells (Table S1); measured and target rate capabilities (Table S2); percentage errors at each C-rate (Table S3) (PDF)
