# Supplementary material for: Battery Electrode Design Process Using Deep Neural Network
Source: ACS Omega. 2026 Sep 28;11(40):61230–40. doi: 10.1021/acsomega.6c05440 (PMC13670279; doi:10.1021/acsomega.6c05440)
Supplement: Supplementary file 1 [file ao6c05440_si_001.pdf]

Supporting Information

Battery Electrode Design Process Using Deep Neural Network

Hyeonghun Park[1,‡], Joosoon Lee[2,‡], Hyungi Song[1], Kyoobin Lee[2,*] and Hyeong-Jin Kim[3,*]

[1] Graduate School of Energy Convergence, Institute of Integrated Technology, Gwangju Institute of Science and Technology, Gwangju 61005, South Korea; bakhh@gm.gist.ac.kr (H.P.); song0610@gm.gist.ac.kr (H.S.)

[2] Department of AI Convergence, Gwangju Institute of Science and Technology, Gwangju 61005, South Korea; joosoon1111@gist.ac.kr (J.L.); kyoobinlee@gist.ac.kr (K.L.)

[3] Department of Chemistry, Gwangju Institute of Science and Technology, Gwangju 61005, South Korea; hjkimc@gist.ac.kr (H.-J.K.)

‡ These authors contributed equally to this work.
* Corresponding authors: kyoobinlee@gist.ac.kr (K.L.); hjkimc@gist.ac.kr (H.-J.K.)

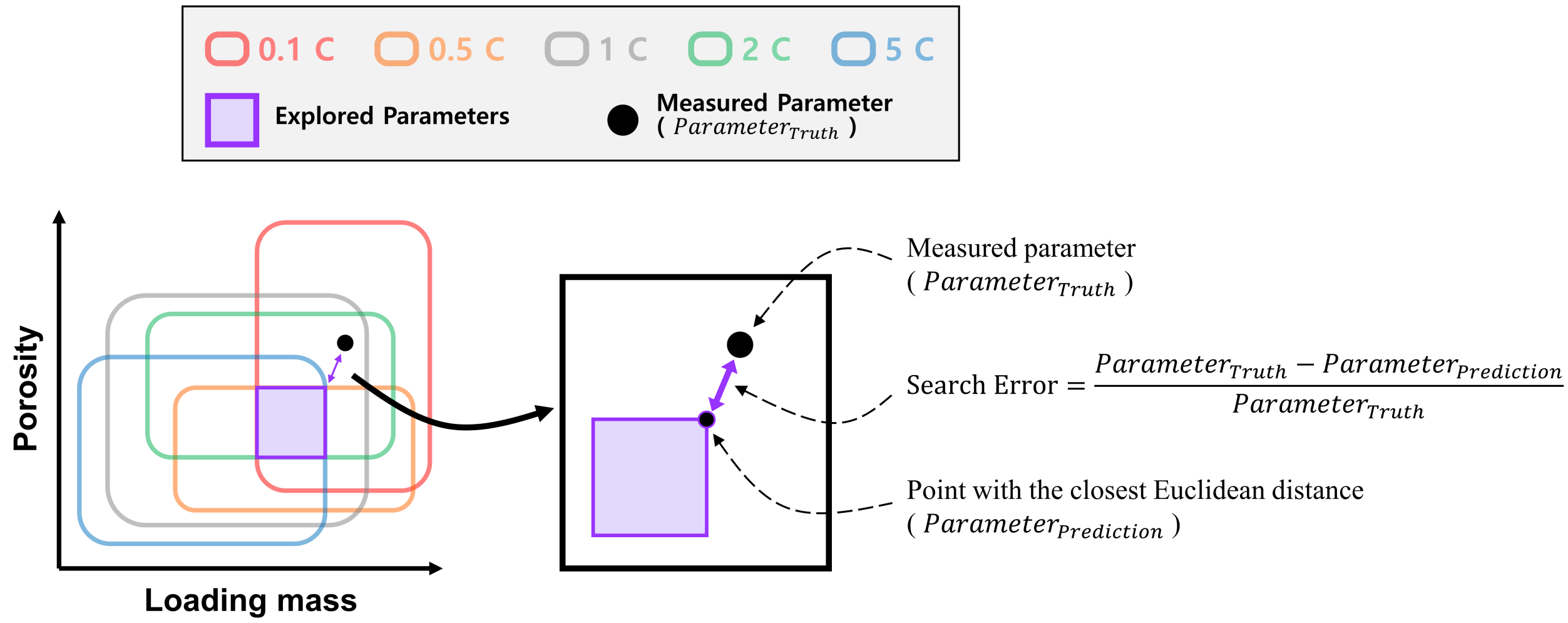

Figure S1: Calculation criteria of the search error.

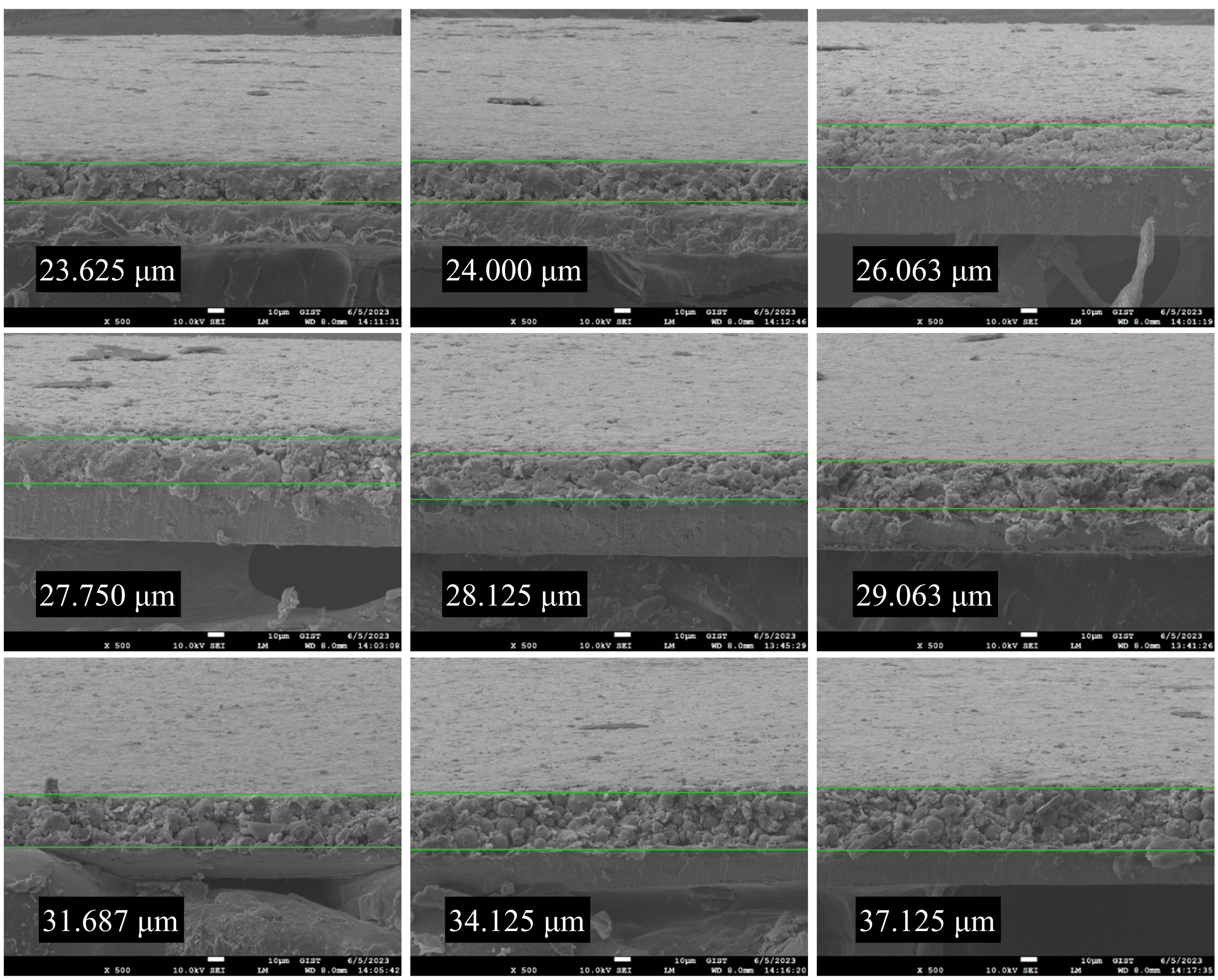

Figure S2: Cross-sectional image of the NMC811 cathode with various designs.

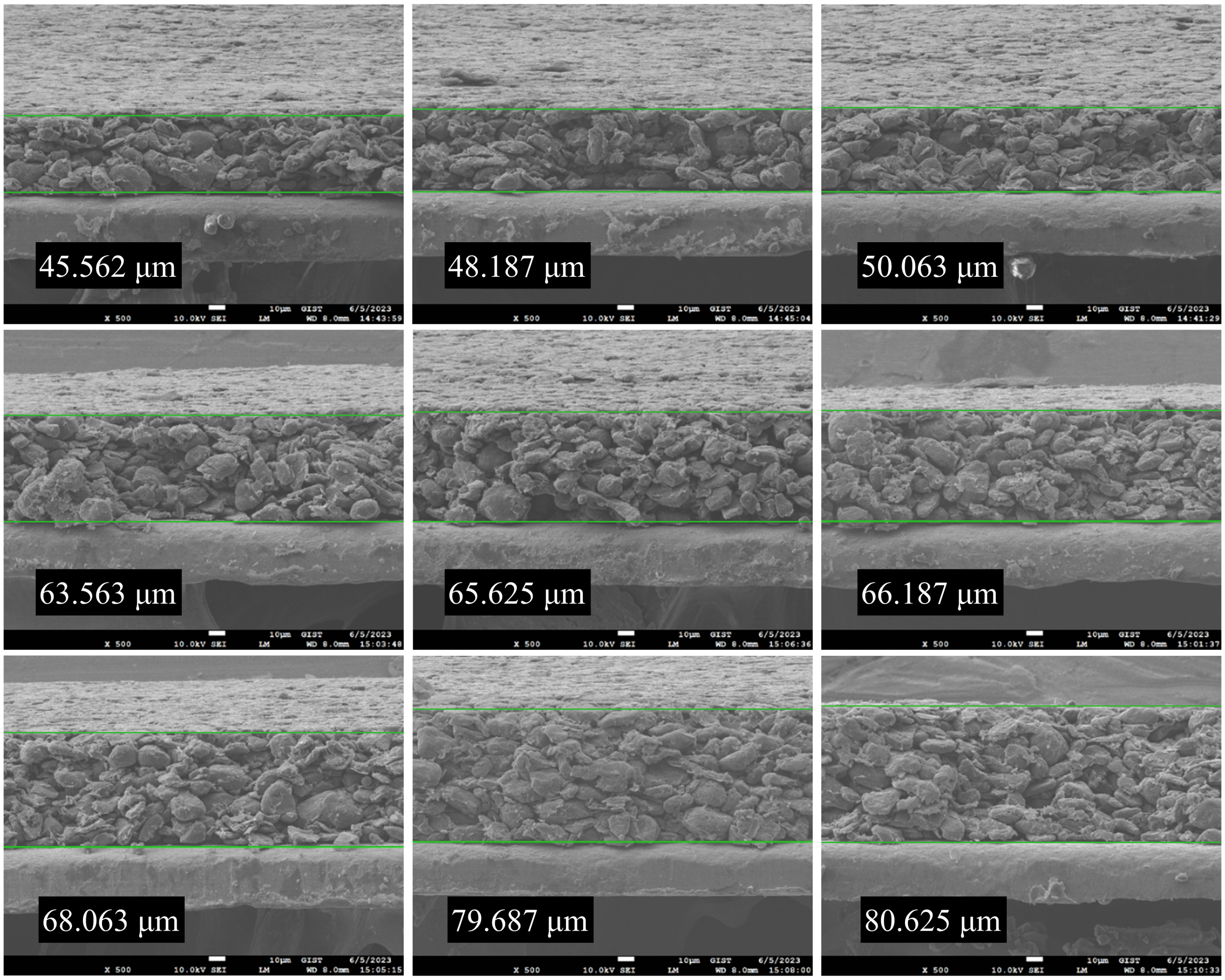

Figure S3: Cross-sectional image of the Gr anode with various designs.

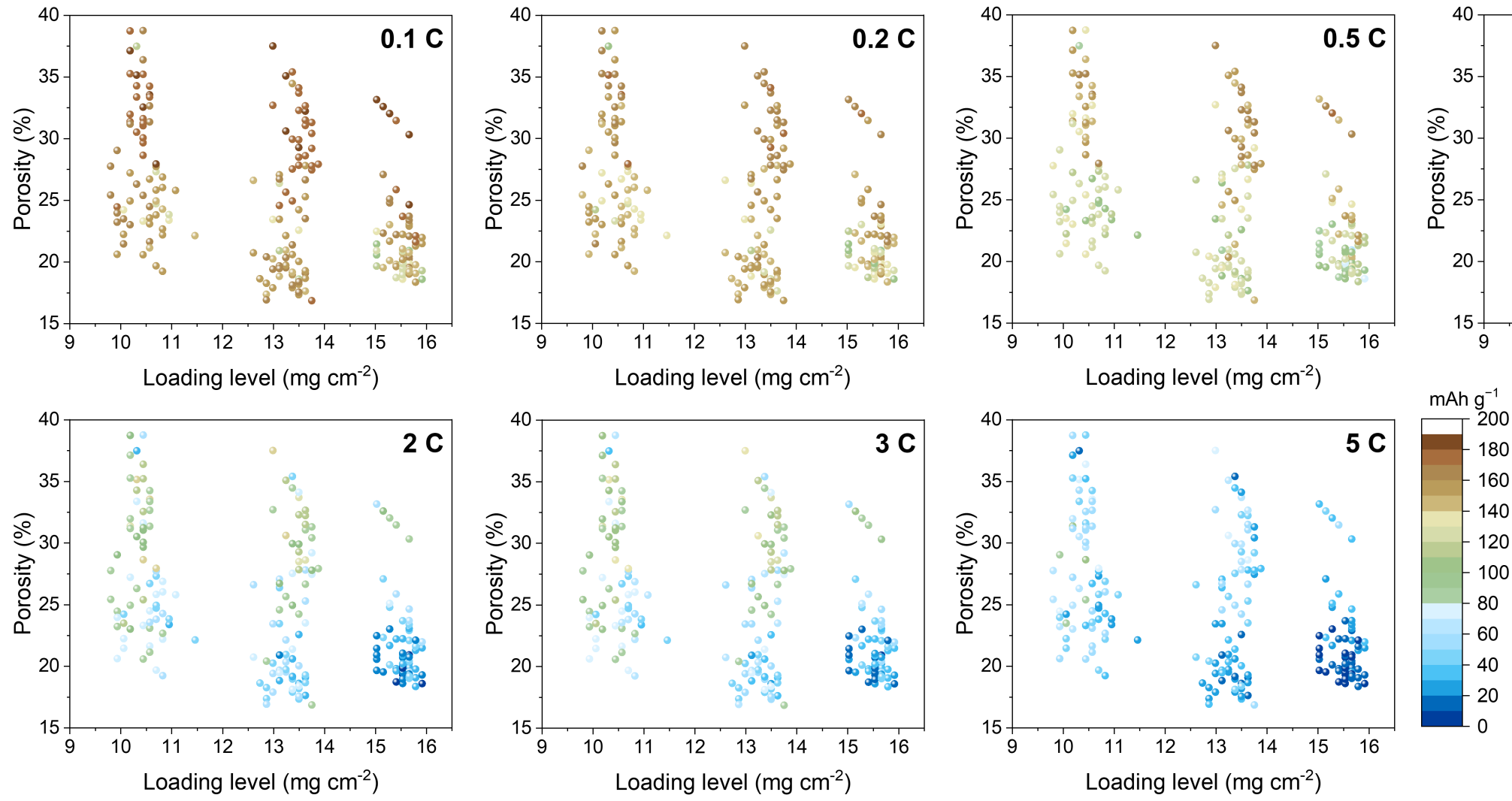

Figure S4: Variations in the specific capacity with different electrode designs.

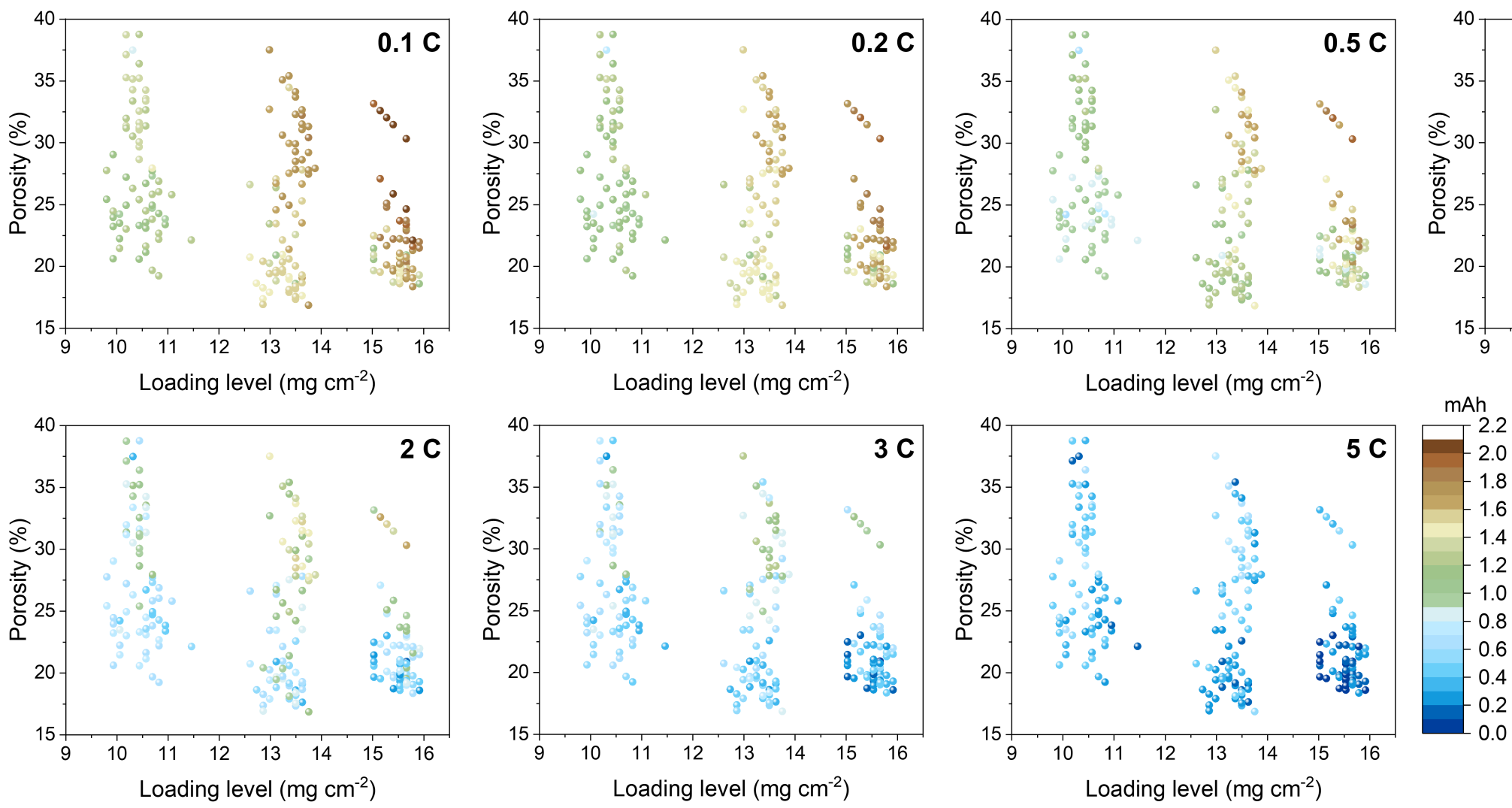

Figure S5: Variations in the capacity with different electrode designs.

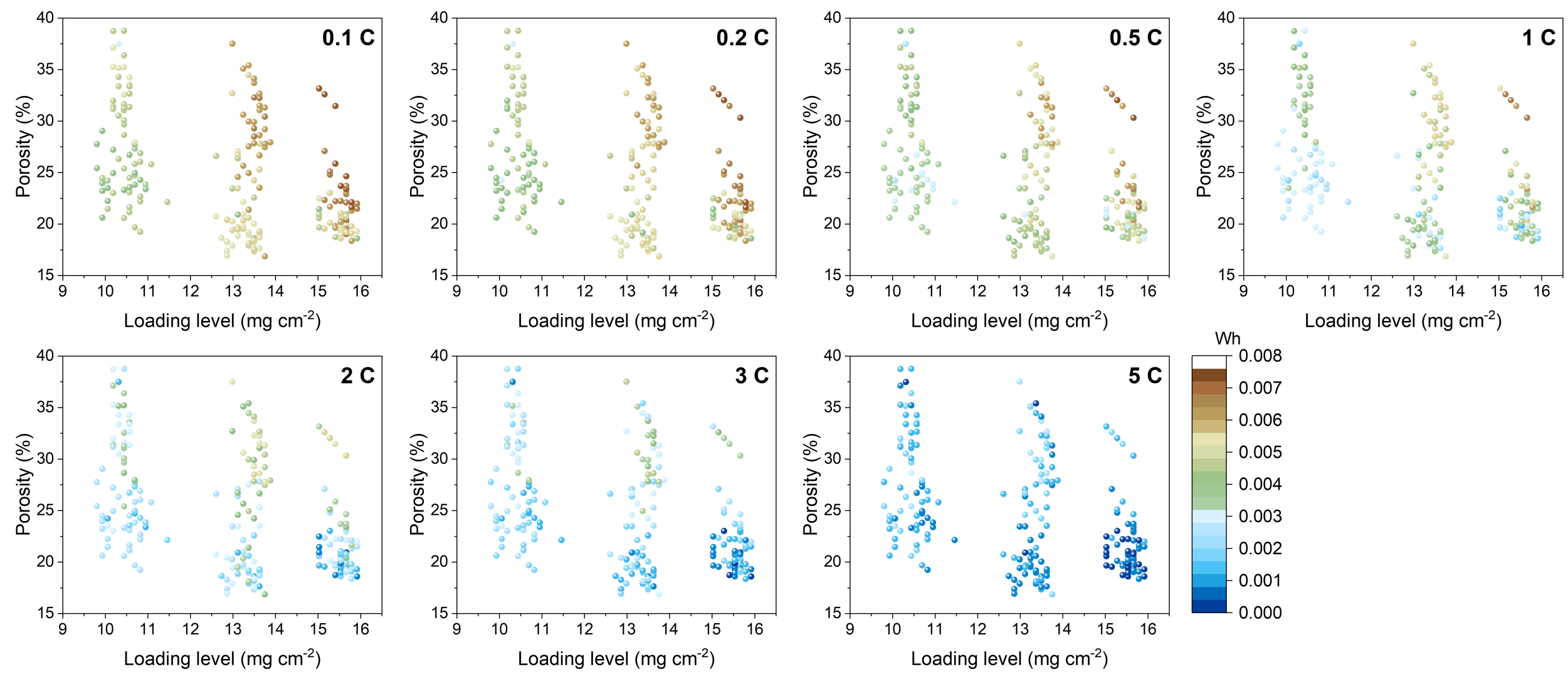

Figure S6: Variations in the energy with different electrode designs.

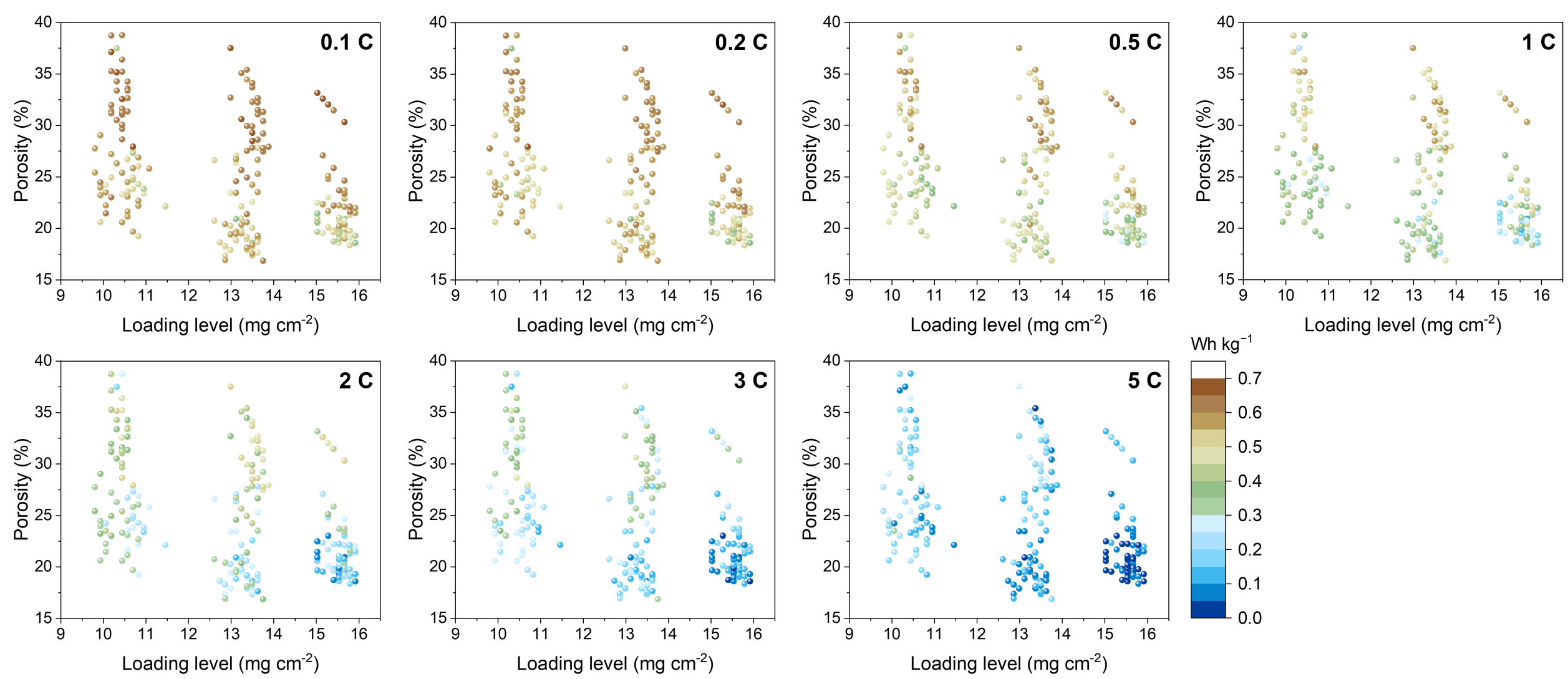

Figure S7: Variations in the energy density with different electrode designs.

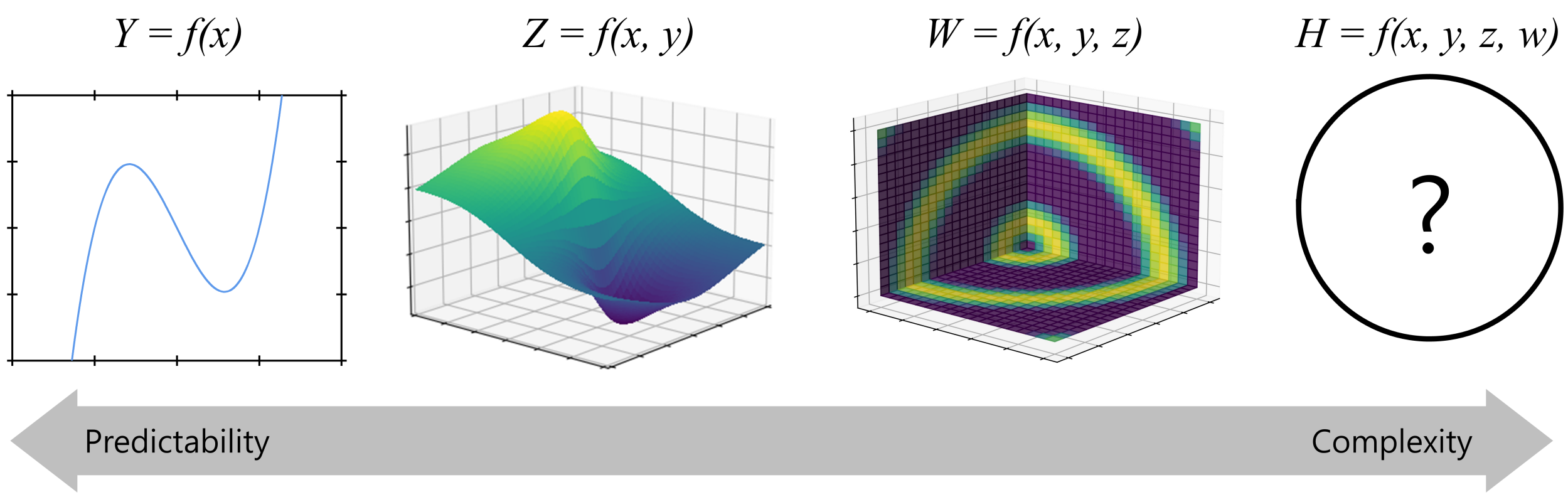

Figure S8: Schematic illustration depicting escalating complexity in plots correlated with the incremental addition of parameters.

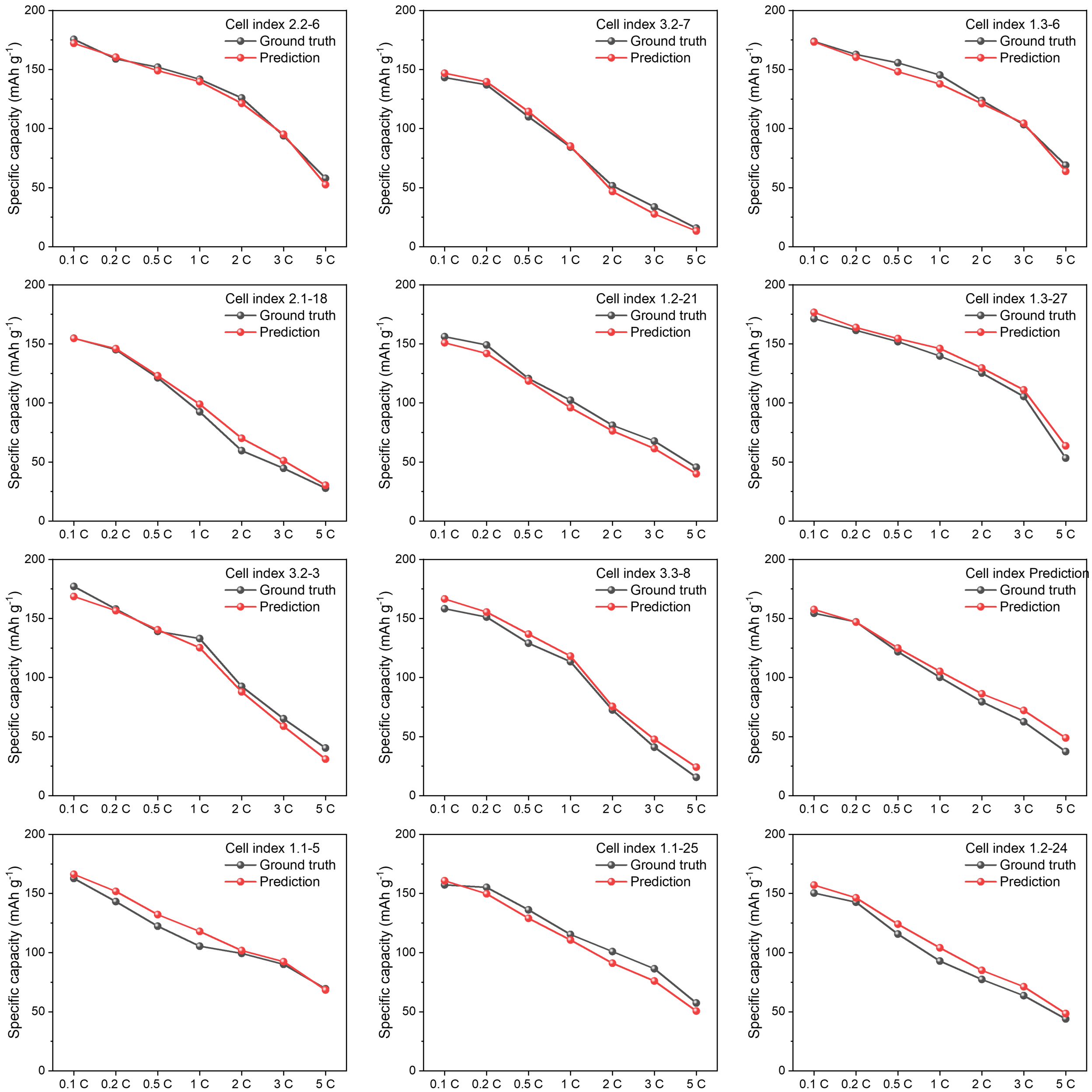

Figure S9: Measured and predicted rate capability of the cell with various design parameters.

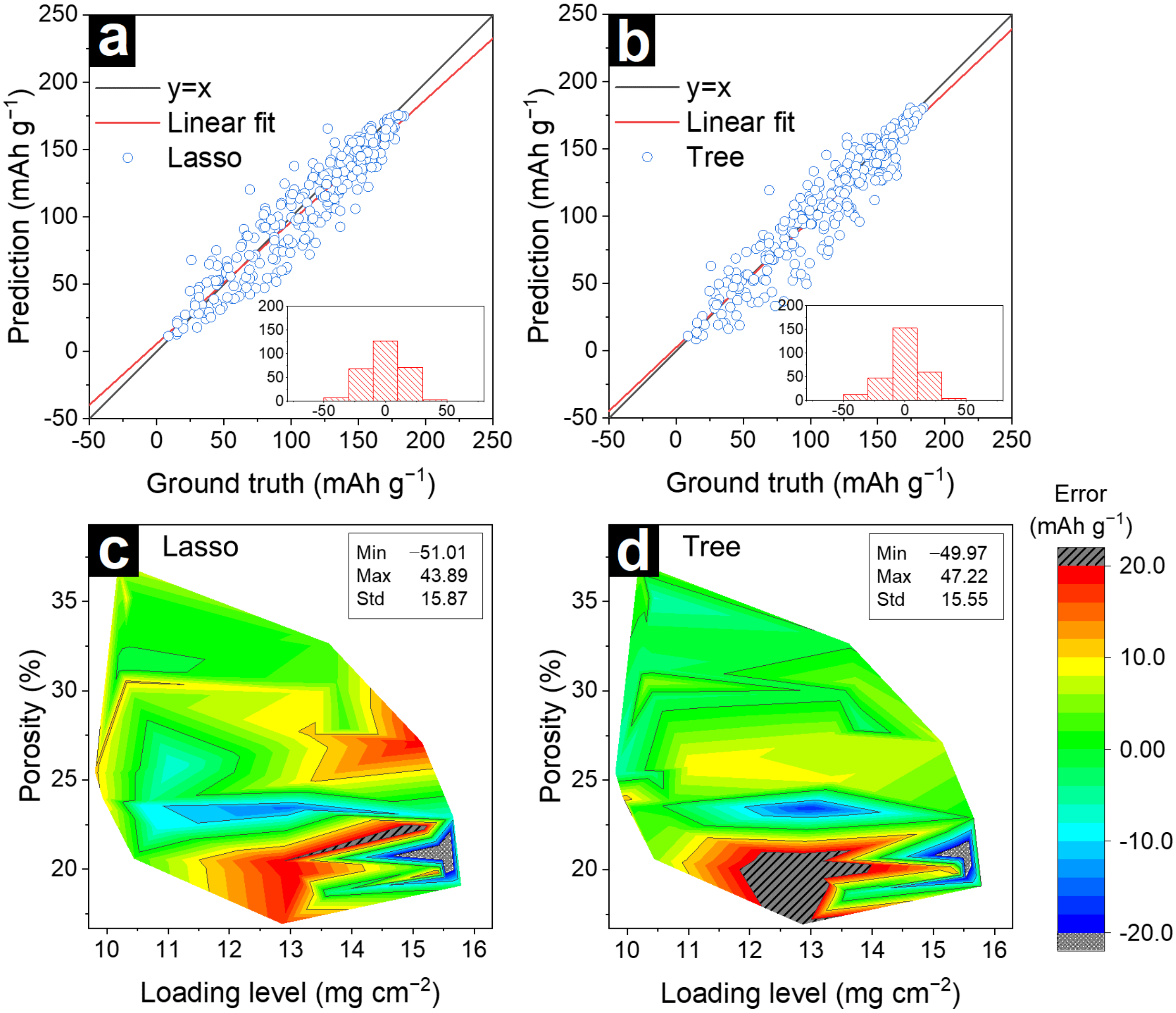

Figure S10: (a–b) Measured vs. predicted capacities for test sets and (c–d) error based on the loading level and porosity obtained using (a, c) lasso regression and (b, d) decision tree regression.

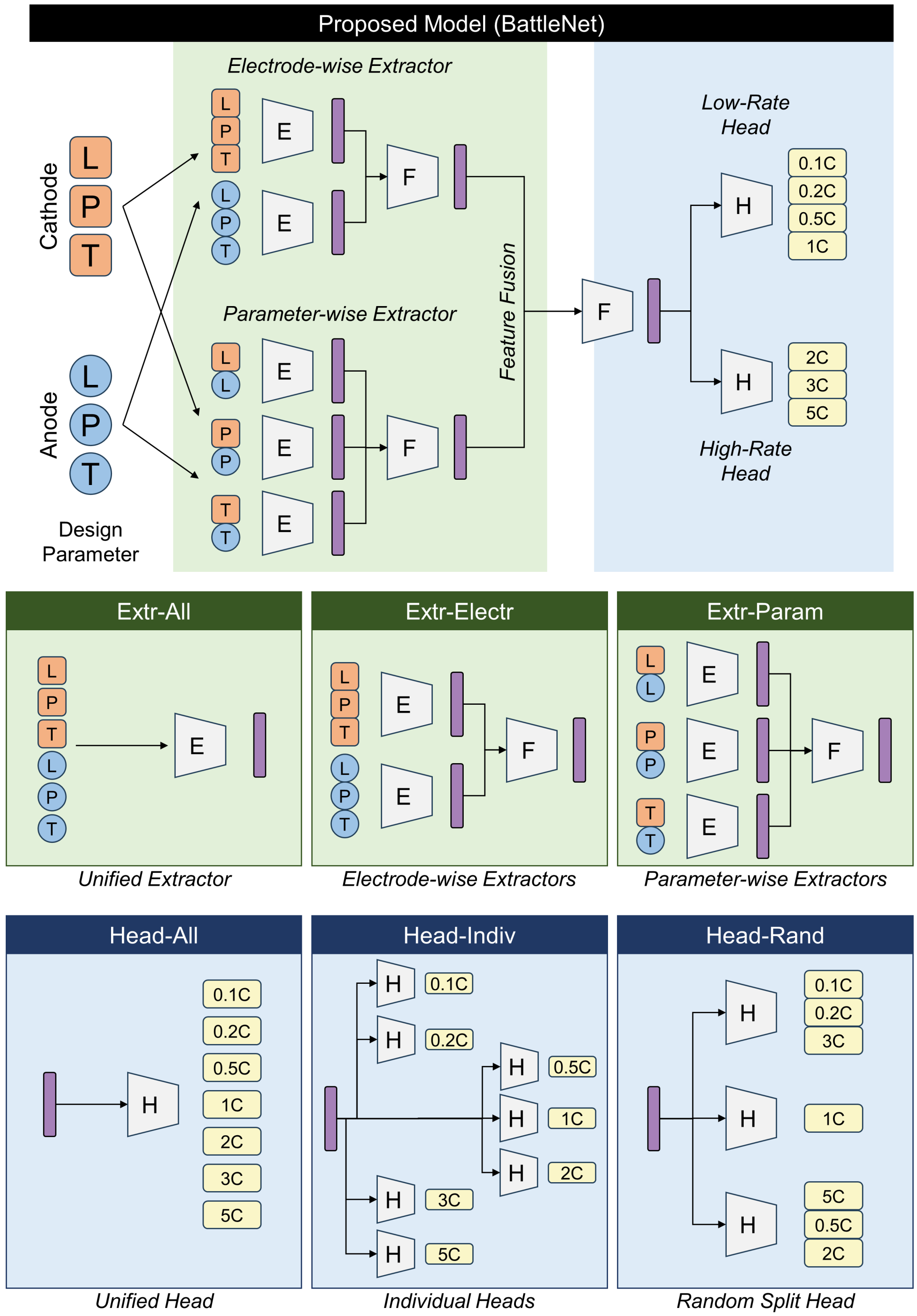

Figure S11: Architectures of the prediction models compared in the ablation study: the proposed BattleNet (top), the feature extractor variants (middle), and the prediction head variants (bottom). E, F, and H denote the feature extractor, feature fusion module, and prediction head, and L, P, and T denote the loading level, porosity, and thickness of the cathode (orange) and anode (blue).

Table S1: Design parameters of cells selected via the proposed framework.

**Cell**	**Cathode**			**Anode**		
	**Loading density (mg cm^{-2})**	**Porosity (%)**	**Thickness (μm)**	**Loading density (mg cm^{-2})**	**Porosity (%)**	**Thickness (μm)**
1	15.3	32.0	53.0	10.1	40.3	75.0
2	15.2	32.6	53.0	9.9	41.4	75.0
3	15.0	33.2	53.0	10.1	40.7	75.0
4	15.4	31.5	53.0	10.1	40.7	75.0

Table S2: Target and measured specific capacity of explored cells. All specific capacities are measured five times and averaged.

**Cell**	**Specific capacity by C-rate (mAh g^{-1})**						
	**0.1C**	**0.2C**	**0.5C**	**1C**	**2C**	**3C**	**5C**
1	187.10	178.67	172.13	165.95	142.03	85.39	33.60
2	183.11	168.38	166.40	163.41	146.52	100.27	44.99
3	181.77	163.90	147.86	127.35	86.87	55.70	32.00
4	179.47	158.93	146.04	146.67	122.84	88.80	51.73
Target	180	160	155	145	125	85	40

Table S3: Percentage error of the cells explored via the proposed framework.

**Cell**	**Percentage error by C-rate (%)**							**Average**
	**0.1C**	**0.2C**	**0.5C**	**1C**	**2C**	**3C**	**5C**	
1	0.0	0.0	0.0	0.0	0.0	0.0	16.0	2.3
2	0.0	0.0	0.0	0.0	0.0	0.0	0.0	0.0
3	0.0	0.0	4.6	12.2	30.5	34.5	20.0	14.5
4	0.3	0.7	5.8	0.0	1.7	0.0	0.0	1.2
Average	0.1	0.2	2.6	3.0	8.1	8.6	9.0	4.5
